# An Oral *Salmonella*-Based Vaccine Inhibits Liver Metastases by Promoting Tumor-Specific T-Cell-Mediated Immunity in Celiac and Portal Lymph Nodes: A Preclinical Study

**DOI:** 10.3389/fimmu.2016.00072

**Published:** 2016-03-01

**Authors:** Alejandrina Vendrell, Claudia Mongini, María José Gravisaco, Andrea Canellada, Agustina Inés Tesone, Juan Carlos Goin, Claudia Inés Waldner

**Affiliations:** ^1^Centro de Estudios Farmacológicos y Botánicos-Consejo Nacional de Investigaciones Científicas y Técnicas (CEFyBO-CONICET), Facultad de Medicina, Universidad de Buenos Aires, Buenos Aires, Argentina; ^2^Instituto de Biotecnología, Instituto Nacional de Tecnología Agropecuaria (INTA), Buenos Aires, Argentina; ^3^Instituto de Estudios de la Inmunidad Humoral Prof. Ricardo A. Margni (IDEHU). Facultad de Farmacia y Bioquímica, Universidad de Buenos Aires, Buenos Aires, Argentina

**Keywords:** *Salmonella* Typhi, vaccine, liver metastases, breast cancer, antitumor CD8^+^ T cells, Th1 immune response, celiac and portal lymph nodes

## Abstract

Primary tumor excision is one of the most widely used therapies of cancer. However, the risk of metastases development still exists following tumor resection. The liver is a common site of metastatic disease for numerous cancers. Breast cancer is one of the most frequent sources of metastases to the liver. The aim of this work was to evaluate the efficacy of the orally administered *Salmonella* Typhi vaccine strain CVD 915 on the development of liver metastases in a mouse model of breast cancer. To this end, one group of BALB/c mice was orogastrically immunized with CVD 915, while another received PBS as a control. After 24 h, mice were injected with LM3 mammary adenocarcinoma cells into the spleen and subjected to splenectomy. This oral *Salmonella*-based vaccine produced an antitumor effect, leading to a decrease in the number and volume of liver metastases. Immunization with *Salmonella* induced an early cellular immune response in mice. This innate stimulation rendered a large production of IFN-γ by intrahepatic immune cells (IHIC) detected within 24 h. An antitumor adaptive immunity was found in the liver and celiac and portal lymph nodes (LDLN) 21 days after oral bacterial inoculation. The antitumor immune response inside the liver was associated with increased CD4^+^ and dendritic cell populations as well as with an inflammatory infiltrate located around liver metastatic nodules. Enlarged levels of inflammatory cytokines (IFN-γ and TNF) were also detected in IHIC. Furthermore, a tumor-specific production of IFN-γ and TNF as well as tumor-specific IFN-γ-producing CD8 T cells (CD8^+^IFN-γ^+^) were found in the celiac and portal lymph nodes of *Salmonella-*treated mice. This study provides first evidence for the involvement of LDLN in the development of an efficient cellular immune response against hepatic tumors, which resulted in the elimination of liver metastases after oral *Salmonella*-based vaccination.

## Introduction

The development of novel cancer treatments is a major subject of study around the globe. More than 90% of cancer-related deaths are due to metastatic disease and not from the primary tumors from which they arise (1). Excision of primary tumors is one of the most widely used cancer treatments. However, the risk of metastases development still exists following tumor resection (2). Breast cancer is the most commonly diagnosed cancer and the second leading cause of cancer-related death in women worldwide. Most deaths from breast cancer are from metastatic disease (3). The liver is a common site of metastatic disease for numerous types of cancer. Lung, colon, pancreatic, mammary, and gastric adenocarcinomas are the most frequent types of carcinoma to metastasize to the liver (4).

Cancer immunotherapy is increasingly acknowledged as an attractive therapeutic option and is now regarded as the fourth anti-cancer treatment (5). The ideal cancer immunotherapy should be tumor specific as well as effective for the residual or metastatic disease with minimal toxicity to normal cells. We have already demonstrated the efficacy of the *Salmonella enterica* serovar Typhi (*S*. Typhi)-attenuated vaccine strain CVD 915 as an immunotherapeutic agent in breast cancer (6) and T-cell lymphoma (7) mouse models. Significant tumor-infiltrating TNF-producing neutrophils along with a decrease in intratumoral IL-10 levels and a reduction of regulatory T cells (Tregs) in draining lymph nodes were observed in both models. These events resulted in a delayed formation of metastases and increased survival times in both studies as well. Furthermore, we found a tumor-specific Th1 immune response in the draining lymph nodes of immunized animals in the mammary adenocarcinoma model. Remarkably, immunotherapy on the metastatic T-cell lymphoma stimulated a tumor-specific T-cell-mediated immunity, which resulted in complete tumor regression in 10% of the treated animals. A long-lasting antigen-specific immune response was induced in most of cured animals, as evidenced by the lack of tumor growth after a rechallenge with the homologous tumor. An additional relevant fact of our findings is that this *S*. Typhi strain is cytotoxic *per se* to murine T-cell lymphoma cells *in vitro*, but innocuous to normal splenocytes.

We have already reported that orogastrically (o.g.) inoculated CVD 915 proves to be a good adjuvant in a prophylactic cancer vaccine against a mouse T-cell lymphoma (8). Based on these findings, we hypothesized that this neoadjuvant cancer therapy could act by activating the pre-existing immune system and breaking immune tolerance to cancer. According to this hypothesis, such an enhanced immune stimulation could promote the generation of effective antitumor responses, which inhibits the development of metastases and enhances the efficacy of the primary cancer therapy.

Despite the inherent tolerogenicity of the liver microenviroment, which protects the organ from chronic inflammation due to intensive influx of antigens from intestinal bacteria (9), the liver is an immunocompetent organ. Indeed, intrahepatic immune cells (IHIC) produce the Th1-type cytokine IFN-γ after a bacterial challenge (10, 11). Our hypothesis suggests that oral administration of the bacterial strain may trigger a systemic proinflammatory immune response leading to a breakdown of the tolerogenic liver environment, thus favoring the detection and rejection of metastatic tumor cells in this organ by an innate immune response and a subsequent tumor-specific immune response. Accordingly, the main goal of this work was to assess the effectiveness of a bacterial-based vaccine on the development of liver metastases in a mouse model of breast cancer. We demonstrated that the orally administered *S*. Typhi CVD 915 promotes an efficient antitumor immune response that results in a decrease in hepatic metastases. Moreover, we found that the recently identified main liver-draining celiac and portal lymph nodes (12, 13) are involved in the immune response against both *Salmonella* and hepatic tumors.

## Materials and Methods

### Cells and Culture Conditions

LM3 is a murine mammary adenocarcinoma cell line syngeneic to BALB/c mice. It was established by Urtreger et al. (14) and kindly provided by Dr. Elisa Bal de Kier Joffé (Research Area, Institute of Oncology Angel H. Roffo, Buenos Aires). Cells derived from passages 58–66 were used throughout this study and were cultured, as previously described (6).

Intrahepatic immune cells, mesenteric lymph nodes (MLN), and liver-draining lymph nodes (LDLN) cells obtained from BALB/c mice were cultured at 37°C in a humidified 5% CO_2_ environment in RPMI supplemented with 2 mM glutamine, 25 mM HEPES, 0.05 mM 2-mercaptoethanol, 100 U/ml penicillin, 100 μg/ml streptomycin, and 10% heat-inactivated fetal calf serum (FCS).

### Bacterial Strain

*Salmonella* Typhi CVD 915 was kindly provided by Dr. Myron Levine (Center for Vaccine Development, University of Maryland School of Medicine, Baltimore, MD, USA). This vaccine strain (15) is a ΔguaBA mutant from *S*. Typhi strain Ty2. Bacteria were routinely cultured and prepared for inoculation, as previously described (6).

### Mice

Eight- to twelve-week-old BALB/c female mice were purchased from the School of Veterinary Sciences, University of Buenos Aires, Argentina. All mice were maintained in an enriched environment and in accordance with institutional animal care and use guidelines. Ethics committee for the care and use of laboratory animals [Comité institucional de cuidado y uso de animales de laboratorio (CICUAL), Facultad de Medicina, UBA] approved the research protocol. Distress and suffering were avoided as much as possible. All procedures were carried out by a veterinarian.

### Treatment and Liver Metastases Tumor Model

Healthy mice were first treated with 50 μl of 10% sodium bicarbonate by o.g. gavage for gastric acid neutralization. After 15 min, they received 200 μl CVD 915 bacterial suspension in sterile PBS (1 × 10^9^ CFU) or PBS alone as a control, also via o.g. Twenty-four hours later, animals were challenged with LM3 tumor cells, using the liver metastases tumor model designed by Lafreniere and Rosenberg (16). Briefly, mice were anesthetized with a combination of Ketamine (100 mg/kg) and Xylazine (10 mg/kg) by intraperitoneal (i.p.) injection. The spleen was inoculated with 1 × 10^5^ LM3 tumor cells through a left subcostal incision, and splenectomy was performed three min later. Animals were housed for 3 weeks until metastases were macroscopically visible. Twenty-four hours or 21 days after treatment animals were sacrificed for sampling. A veterinarian routinely monitored sudden deaths and clinical signs of discomfort or illness in all mice throughout the experiments, such as weight loss, apathy, trembling, restricted motility, vomits, diarrhea, rash, and swelling.

Mice were weighted before treatment and at sacrifice. For sampling, they were anesthetized with Ketamine/Xylazine (i.p.) and bled by heart puncture with a heparinized syringe. Livers were perfused with cold PBS via the portal vein and then removed, weighted, and processed for the isolation of IHIC. Spleen, MLN, and/or LDLN (portal and celiac nodes) were removed and disaggregated into a single-cell suspension. Celiac and portal lymph nodes were identified anatomically as described by Zheng et al. (13) and sampled.

Hepatic metastases were measured and quantified using a stereo zoom microscope (loupe) (4×) (16). The hepatic tumor volume was calculated as the sum of the volumes of all nodules, which were estimated using the two-dimensional mathematical formula for ellipsoidal tumors: *V* = π/6 × *f* × (length × width)^3/2^ (17).

The liver metastases model used has an effectiveness of ~88% and does not allow discrimination between mice that failed to develop metastases from those eventually cured by the treatment. Thus, only those animals showing macroscopically visible metastases 20 days after the challenge were included in further analysis.

### Quantification of *Salmonella* in Liver

Balb/c mice were treated with CVD 915 via o.g. route, as described previously. One, four, eight, or twenty-four hours after the bacterial inoculation, mice were euthanized and livers aseptically removed and homogenized in distilled water. Samples of homogenized livers were seeded in agar plates for CFU determination, as previously described (6).

### Intrahepatic Immune Cell Isolation

Intrahepatic immune cells were isolated by mechanical disruption followed by Percoll density separation as described by Blom et al. (18), with minor modifications. Briefly, livers were disaggregated through a nylon cell strainer, using a syringe plunger, and then suspended in 50 ml of RPMI 10% FCS. This suspension was centrifuged at 60 × *g* for 1 min at room temperature without brake. The resulting supernatant was centrifuged at 480 × *g* with brake for 8 min at room temperature. The pellet obtained was resuspended in 5 ml of Percoll 33% in PBS with heparin (100 U/ml) and centrifuged at 850 × *g* for an additional 30 min at room temperature without brake. The resulting pellet, containing the IHIC and contaminant erythrocytes, was subjected to hypoosmotic lysis and then washed with complete medium. Cells were then resuspended in complete medium for cell surface phenotyping by flow cytometry or functional analysis.

### Immunofluorescence Staining and Flow Cytometric Analysis

Blood, spleen, MLN, LDLN, and IHIC were assayed for phenotypic analysis by immunofluorescence and flow cytometry, using rat monoclonal antibodies against mouse leukocyte antigens, as follows: PE anti-CD8, FITC anti-CD4, PE anti-B220, PE anti-CD11c, biotin anti-CD49, PE anti-Ly-6G (Gr1), and biotin anti-MHC II (*e*Bioscience, San Diego, CA, USA). APC- or PE-conjugated streptavidin and isotype-matched controls were used as required. Immunofluorescence surface staining and flow cytometric analysis were performed, as previously described (6). The neutrophil population was defined as that expressing the highest level of Gr1 and gated as shown in Figure S1 in Supplementary Material. The dendritic cell (DC) subset was defined as that expressing CD11c gated as shown in Figure S1 in Supplementary Material. Lymphocytes were gated as shown in Figure S1 in Supplementary Material.

IFN-γ-production by tumor-specific CD8^+^ T cells was measured by intracellular staining, as previously described (6). IHIC and LDLN cells, obtained from mice after 20 days of tumor challenge, were cultured for 96 h in the presence or absence of LM3 cells (at a ratio 10:1). Thereafter, cells were activated for 4 h with PMA (20 ng/ml) and ionomycin (500 ng/ml, both from Sigma, St. Louis, MO, USA) in the presence of 2 μg/ml Brefeldin A (BFA, *e*Bioscience), and then labeled with fluorescent antibodies, as previously described. Samples were processed using a BD FACSCalibur or a BD Accuri C6 flow cytometer (BD Biosciences, San Jose, CA, USA). Multicolor flow cytometric analysis was performed using the Flowing Software 2.5.1 (Perttu Thero, Turku, Finland).

### Measurement of Cytokines by ELISA

Intrahepatic immune cells and LDLN cells (0.5 × 10^6^ and 1 × 10^6^/well, respectively) obtained from treated and control mice were cultured separately in 96-well plates in the presence or absence of LM3 tumor cells at a 10:1 ratio. Supernatants were collected 72- or 96-h later, and cytokines (IFN-γ, TNF, and IL-2) were measured by ELISA (eBioscience) following the manufacturer’s instructions. The detection limits were 30, 4, and 4 pg/ml, respectively.

### Histopathological Analysis

Twenty-one days after treatment, *S*. Typhi-treated and control mice were euthanized. Livers were removed and fixed with 10% buffered formalin before paraffin embedding. Hematoxylin and eosin (H&E) staining of paraffin-embedded sections was performed, as previously described (19). Organs were examined for the presence of metastases and leukocyte infiltrates.

### Statistical Analysis

All data are shown as mean ± SEM. For comparisons between groups, unpaired Student’s *t*-test was used. For comparisons between more than two groups, an ANOVA followed by a post-ANOVA Tukey’s multiple comparison test was performed. When data were not normally distributed, the Mann–Whitney test was used for comparison between two groups. Differences were considered significant when *p* < 0.05 for all comparisons. The statistical analysis was performed using Prism 5.0 software (GraphPad, La Jolla, CA, USA).

## Results

### Oral *S*. Typhi Does Not Colonize Mouse Liver

Orally delivered attenuated *Salmonella* Typhimurium has been shown to infect mouse liver (20). However, there have been no studies investigating liver colonization by attenuated *S*. Typhi strains. In mice inoculated orally with *S*. Typhi vaccine strain CVD 915, we found no vaccine organisms in liver tissue removed 1, 4, 8, and 24 h post vaccination.

### Oral Vaccination with CVD 915 in Naive Mice Induces an Early Immune Response in the Liver and LDLN

To determine whether oral immunization with *S*. Typhi could elicit an efficient immune response in the mouse liver, we inoculated orally naive mice with 10^9^ CFU of CVD 915. Twenty-four hours later, mice were sacrificed and blood, spleen, liver, and LDLN were analyzed.

**Figure 1:**
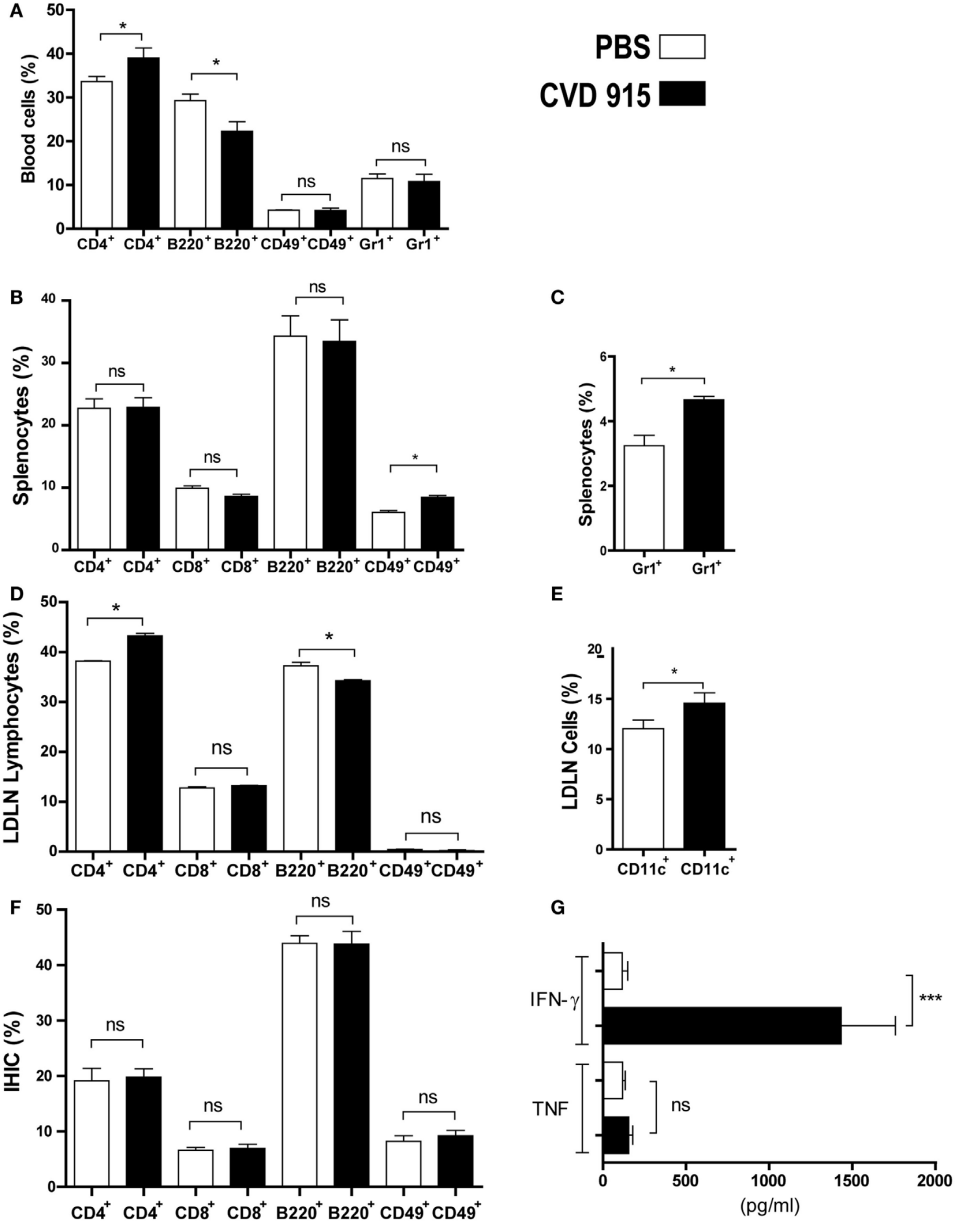
**Early immune response with IFN-γ production in liver of naive mice after oral *Salmonella* administration**. BALB/c mice were immunized with CVD 915 via o.g. or PBS as a control. After 24 h, animals were sacrificed for sampling. **(A–F)** Cell phenotype of individual mice was analyzed by flow cytometry (*n* = 3–13). **(A)** Percentage of blood cells. **(B)** Percentage of splenic lymphocytes. **(C)** Percentage of splenic neutrophils. **(D)** Percentage of lymphocytes from liver-draining lymph nodes (LDLN). **(A–D)** *p < 0.05; ns, not statistically significant. Data are from one experiment representative of three to five. **(E)** Percentage of CD11c^+^ cells between LDLN cells, gated on DC region as defined in Section “Materials and Methods.” *p < 0.05. **(F)** Percentage of lymphocytes from intrahepatic immune cells (IHIC). ns, not statistically significant. **(E,F)** Data are from three experiments. **(G)** IFN-γ and TNF levels produced by IHIC measured by ELISA. The limits of detection were 30 and 4 pg/ml, respectively. ***p < 0.001; ns = not statistically significant (*n* = 9). Data are from two experiments.

We found an increased frequency of circulating CD4^+^ T cells and a decreased population of B220^+^ B cells from *Salmonella*-immunized mice as compared with PBS-inoculated control ones (Figure 1A). Furthermore, enhanced frequencies of innate immune cells, NK (CD49^+^) and neutrophils (Gr1_+_), were detected in the spleen (Figures 1B,C, respectively).

We next assessed the involvement of the main LDLN in the immune response after oral immunization with *Salmonella*. The frequency of CD4^+^ T cells was enhanced at the expense of decreased B220^+^ B cells in LDLN from *Salmonella*-immunized mice with respect to control ones (Figure 1D). Moreover, the percentage of DC (CD11c^+^) was also increased in treated mice as compared with control animals (Figure 1E).

The phenotypic analysis of IHIC populations from treated mice did not reveal any variations in the proportion of CD4^+^, CD8^+^, B220^+^, or CD49^+^ lymphocytes as compared with control mice, as shown in Figure 1F. However, the measurement of cytokine production by IHIC revealed a significant difference between both groups. IHIC were cultured *ex vivo* for 72 h without additional stimulation. Then, cytokines synthesized by these cells were measured in the culture supernatant by ELISA (Figure 1G). An increase in IFN-γ levels produced by the IHIC of more than 10-fold was detected in *Salmonella*-treated mice, as compared with control animals, whereas no differences were found in TNF levels. This result indicates a strong innate IFN-γ production in the liver, which is triggered by the oral-administered *Salmonella*.

Taken together, these results confirm that the oral vaccination with CVD 915 was capable of inducing a systemic (in blood and spleen) and local (in liver and LDLN) innate immune response.

### Oral *Salmonella-*Based Immunotherapy Reduces the Hepatic LM3 Tumor Burden and Proves to Be Well Tolerated

**Figure 2:**
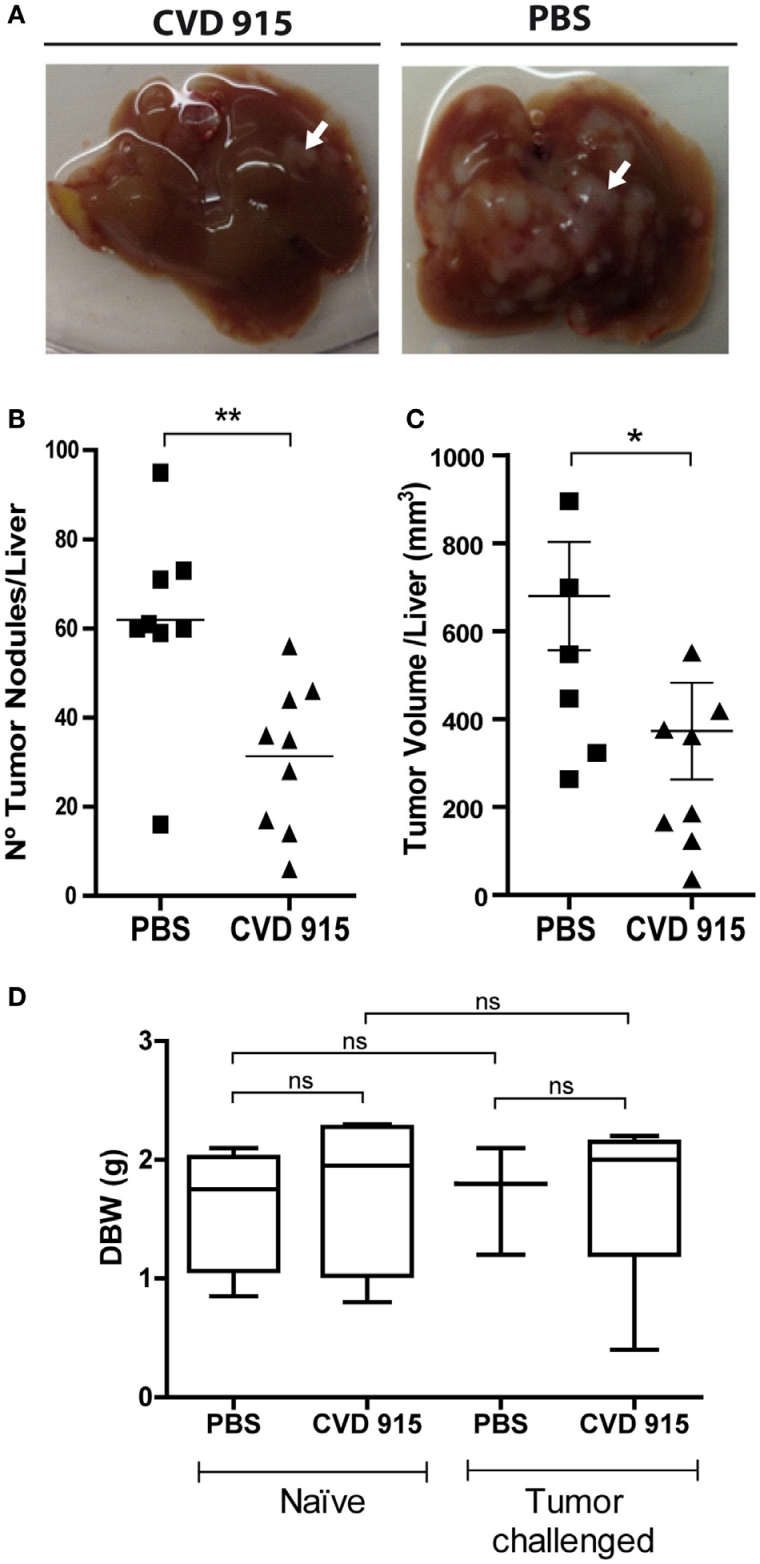
**Anti-metastatic effects of the oral *Salmonella* vaccine CVD 915**. Twenty-one days after treatment with CVD 915 or PBS, livers from tumor-bearing mice were removed. **(A)** Representative images of one liver from each group are shown. Arrows indicate a representative tumor nodule **(B)** Number of superficial tumor nodules per liver. **(C)** Liver tumor volumes. **(B,C)** Dots represent the tumor number or volume of each mouse. *p < 0.05, **p < 0.01 (*n* = 8–9). Data are from two experiments representative of four. **(D)** Body weight gain of naive and tumor-challenged mice. Mice were weighed before (day 0) and 21 days after treatment. Box and whiskers represent the differential body weight (DBW) of each group. ns, not statistically significant (*n* = 3–5). Data are from one experiment representative of three.

To assess the incidence of liver metastases following oral vaccination with CVD 915, mice were orally immunized with *Salmonella* or PBS. Twenty-four hours later, LM3 tumor cells were inoculated using the liver metastases mouse model explained above. The prophylactic efficacy of bacterial administration on the development of liver metastases was evaluated 20 days later, by counting and measuring liver tumor nodules. A significant decrease in the hepatic tumor burden was found in *Salmonella*-treated mice (Figure 2A). Indeed, livers from treated animals showed a 50% reduction in the number of tumor nodules (Figure 2B) and a 45% decrease in tumor volume, as compared with PBS-treated mice (Figure 2C). Furthermore, livers from non-treated animals were ~15% heavier than those from *Salmonella*-treated ones (mean ± SEM) (1.43 ± 0.10 vs. 1.22 ± 0.06 g, respectively; *p* = 0.04), indicating that oral *Salmonella* promotes tumor mass reduction. These results show a strong therapeutic efficacy of this bacteria-based vaccine in preventing the development of liver metastases.

Reduction in body weight gain is a sensitive indicator of toxicity in rodent studies (21). In order to assess safety and tolerability of the oral treatment with *Salmonella*, tumor-challenged and naive animals were weighted, before and after treatment, and side effects were monitored. Mice from all groups gained almost 2 g in 3 weeks. There was no disparity in body weights between control and treated mice, either inoculated or not with LM3 cells (Figure 2D). Furthermore, no adverse effects were noted during the experiment and all animals appeared to be healthy upon clinical observation. These data suggest that oral *Salmonella-*based vaccine was well tolerated both in naive and tumor-bearing animals.

### Oral *Salmonella* Achieves an Antitumor Therapeutic Effect by Triggering a Cell-Mediated Immune Response in the Liver and LDLN

**Figure 3:**
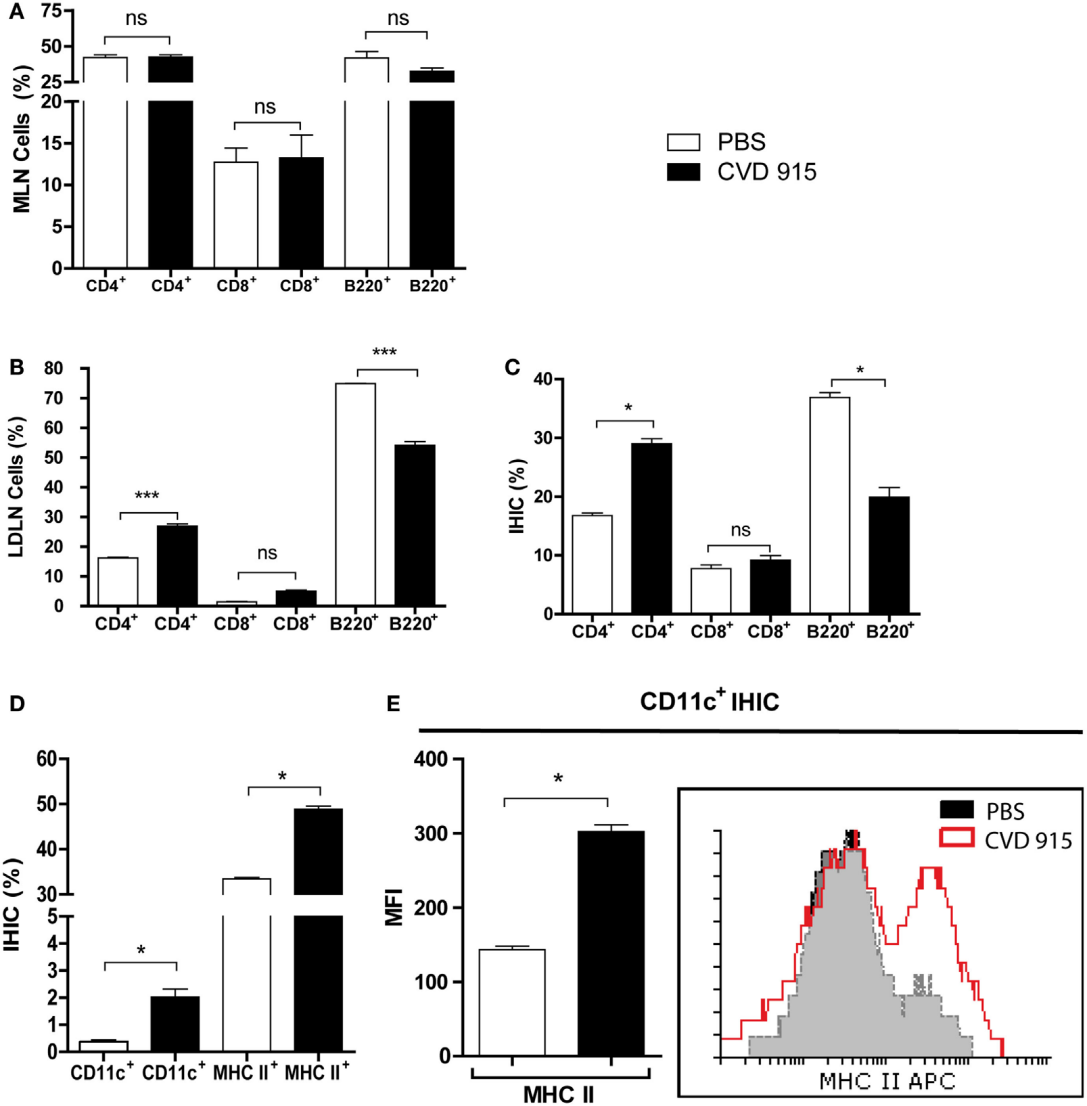
**Enhance cellular immune response in the liver and LNDN after cancer treatment with oral *Salmonella***. Twenty-one days after treatment with CVD 915 or PBS, livers, mesenteric lymph nodes (MLN) and liver draining lymph nodes (LDLN) from tumor-bearing mice were analyzed by flow cytometry. **(A)** Percentages of CD4^+^, CD8^+^, and B220^+^ lymphocytes from MLN. **(B)** Percentages of CD4^+^, CD8^+^, and B220^+^ lymphocytes from LDLN. **(C)** Percentages of CD4^+^, CD8^+^, and B220^+^ lymphocytes from intrahepatic immune cells (IHIC). **(D)** Percentages of CD11c^+^ and MHC II^+^ cells between the IHIC. **(E)** Representative histograms showing expression of MHC class II on gated CD11c^+^ DC from IHIC. Bar graph represents the median fluorescence intensity (MFI) of MHC II expression on CD11c^+^ cells. **(A–D)** ns, not statistically significant, *p < 0.05, ***p < 0.0001. All data shown are mean values of three pooled samples from four to five animals per group and are representative of one to four experiments.

Twenty-one days after treatment, the liver, MLN, and LDLN were removed and leukocyte populations from the organs were characterized. The phenotypic analysis of MLN populations from treated mice did not reveal any differences in the percentages of CD4^+^, CD8^+^ or B220^+^ lymphocytes as compared with control mice (*p* > 0.05), as shown in Figure 3A. This result indicated that MLN are not involved in the antitumor cellular immune response against liver metastases generated after oral *Salmonella*-based treatment. We next examined whether the liver and LDLN are involved in the immune mechanism underlying the antitumoral effects of this oral vaccine. An increased frequency of CD4^+^ T cells at the expense of decreased B cells (B220^+^) was detected in CVD 915-treated mice when compared to controls (Figure 3B). Interestingly, a similar pattern was found in IHIC, with an increased frequency of CD4^+^ T cells and a decrease in the B220^+^ cell population (Figure 3C). No significant differences in the frequencies of CD8^+^ T cells were found between both groups of mice. In addition, the percentages of CD11c^+^ and MHC II^+^ expressing-cells were increased in the liver (Figure 3D). Moreover, DC (CD11c^+^ cells) from treated-mice expressed higher levels of MHC II molecules than DC from controls (Figure 3E). These results indicate that oral *Salmonella*-based vaccine promotes an antitumor cell-mediated immune response in the liver and LDLN.

### Oral *Salmonella* Induces an Antitumor Th1-Type Cellular Immune Response in the Liver and LDLN

**Figure 4:**
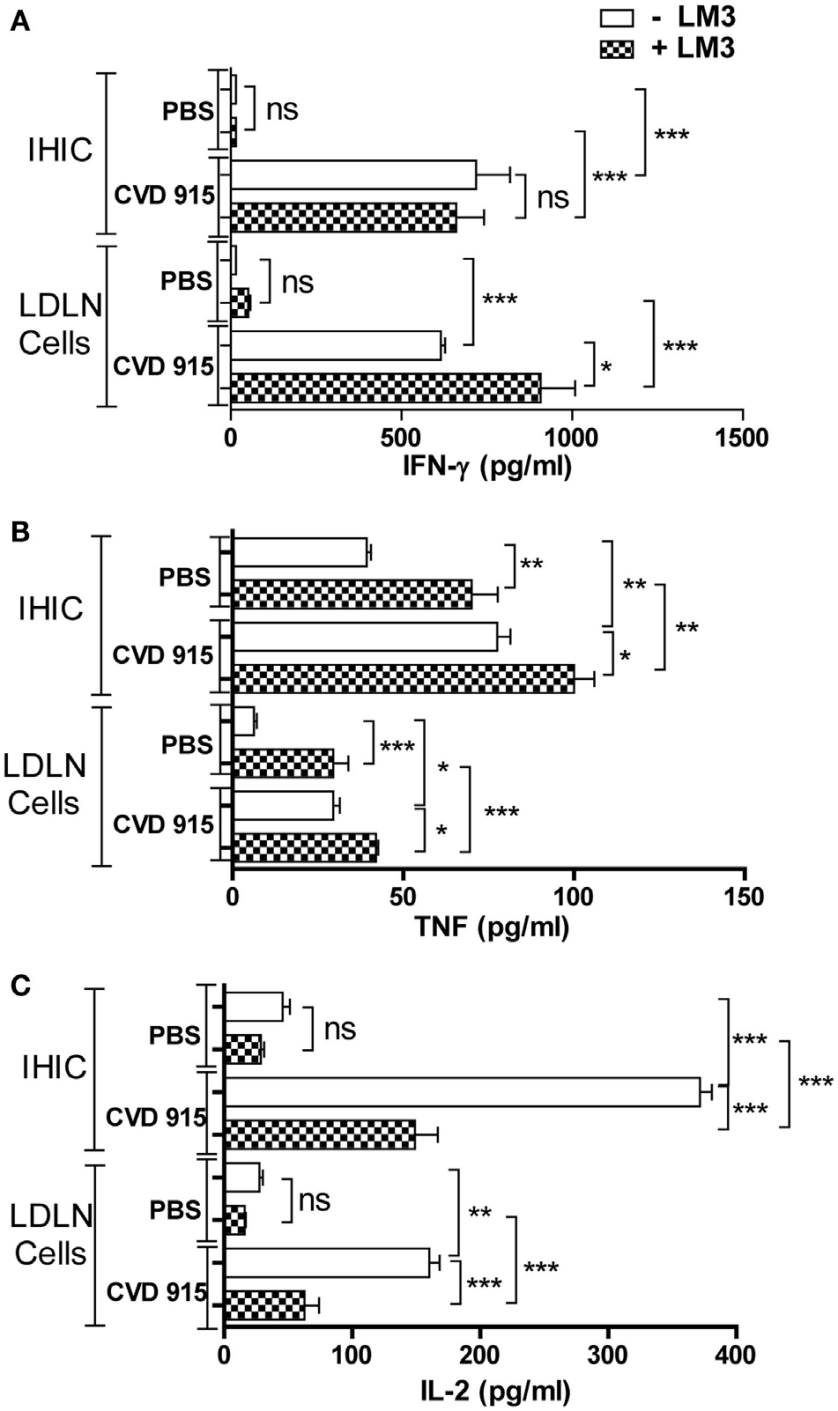
**Antitumor Th1-type cytokine production in the liver and liver-draining lymph nodes (LDLN) from *Salmonella*-treated mice**. Twenty-one days after treatment with CVD 915 or PBS, livers and LDLN from tumor-bearing mice were removed. Cytokine production was measured by ELISA in 96 h culture supernatants of intrahepatic immune cells (IHIC) and LDLN cells following exposure to LM3 tumor or not. **(A)** IFN-γ, **(B)** TNF, and **(C)** IL-2. The limits of detection were 30, 4, and 4 pg/ml, respectively. Samples from four to five animals per group were pooled. Data shown are mean values of triplicate or quadruplicate cultures and are representative of two independent experiments. ns, not statistically significant, *p < 0.05, **p < 0.001, ***p < 0.0001.

Immune analysis was performed 21 days after immunization. Primary cultures from IHIC or LDLN cells were exposed (or not) to LM3 cells. Then, levels of IL-2, TNF, and IFN-γ were determined in culture supernatants. In cultures unexposed to tumor cells, enhanced levels of these three cytokines were found in IHIC and LDLN cultures from *Salmonella*-treated mice, as compared with those from control PBS-treated mice (Figures 4A–C). When LDLN cells from *Salmonella*-treated mice were exposed to tumor cells, the levels of IFN-γ and TNF increased, whereas the levels of IL-2 decreased with respect to the unexposed culture from the same group (Figures 4A–C). When IHIC from bacterial-treated animals were cultured with tumor cells, the level of TNF enlarged, whereas the concentration of IL-2 decreased with respect to the unexposed culture from the same group. Nevertheless, IFN-γ levels generated by IHIC from bacteria-treated animals did not increase in the presence of LM3 cells (Figure 4A). These results suggest that a tumor-specific Th1-type immune response is elicited in the liver and its associated draining lymph nodes in *Salmonella*-treated mice.

### Oral *Salmonella* Elicits Tumor-Specific CD8^+^IFN-γ^+^ Effector T Cells in LDLN

**Figure 5:**
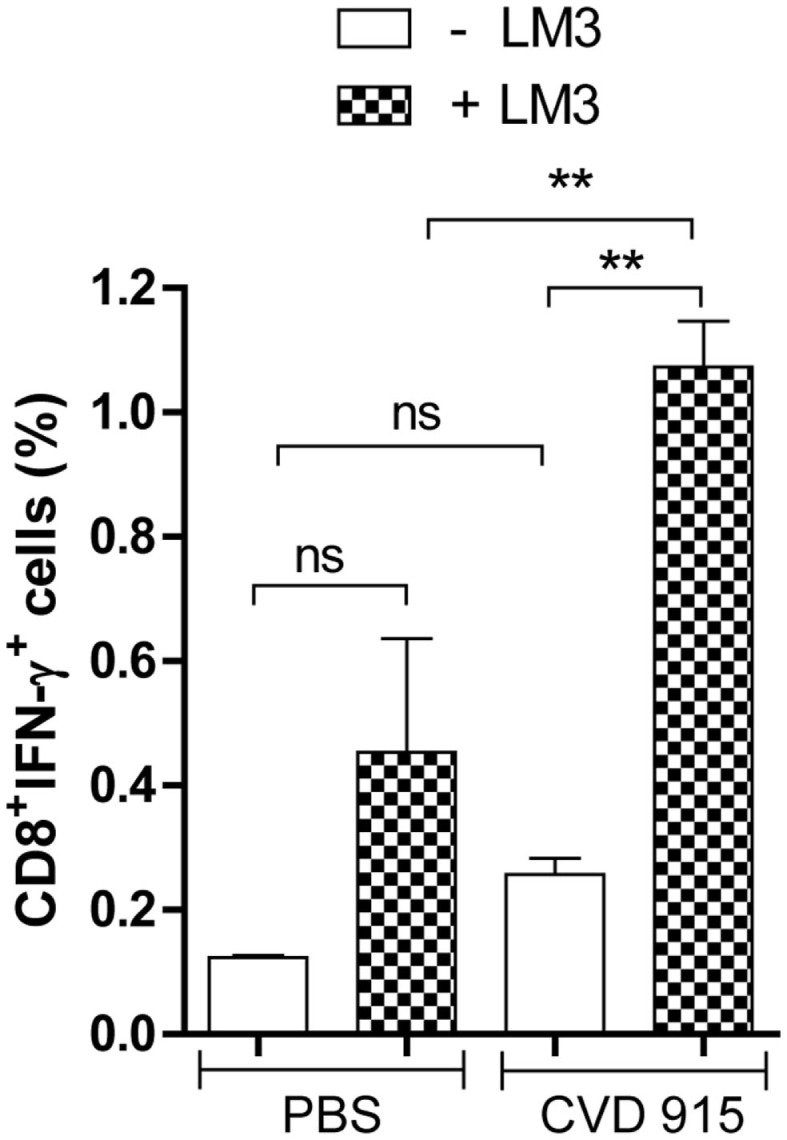
**Tumor-specific IFN-γ-producing CD8^+^ cells in liver-draining lymph nodes (LDLN) after oral *Salmonella*-based treatment**. Twenty-one days after treatment with CVD 915 or PBS, LDLN from tumor-bearing mice were removed. Samples from three animals per group were pooled. LDLN cells were stimulated or not with LM3 tumor cells for 96 h. Then, CD8^+^ surface expression and intracellular IFN-γ production were measured by flow cytometry as indicated in Section “Materials and Methods.” Bars represent the mean ± SEM percentage of CD8^+^IFN-γ^+^ T cells per culture of LDLN cells. **p < 0.01 (*n* = 6–10).

After a 96-h coculture of LDLN cells from PBS- or *Salmonella*-treated mice with (or without) LM3 tumor cells, immune cells were immunocharacterized and stained for intracytoplasmic IFN-γ. An enlarged percentage of CD8^+^IFN-γ^+^ T-cells was found in LDLN from bacteria-treated mice that had been cultured with tumor cells, as compared with non-treated controls (Figure 5). Interestingly, in the *Salmonella*-treated group, the IFN-γ production by CD8 T cells was higher after incubation with LM3 tumor cells. Representative dot plots showing percentage of CD8^+^IFN-γ^+^ T-cells are available in the Figure S2 in Supplementary Material. These results indicate that oral immunization with *Salmonella* stimulates a tumor-specific immune response that is capable of generating effector CD8^+^ T cells in LDLN.

### Oral *Salmonella*-Based Immunotherapy Induces Leukocytic Infiltration in the Liver, Mainly around Metastatic Nodules

**Figure 6:**
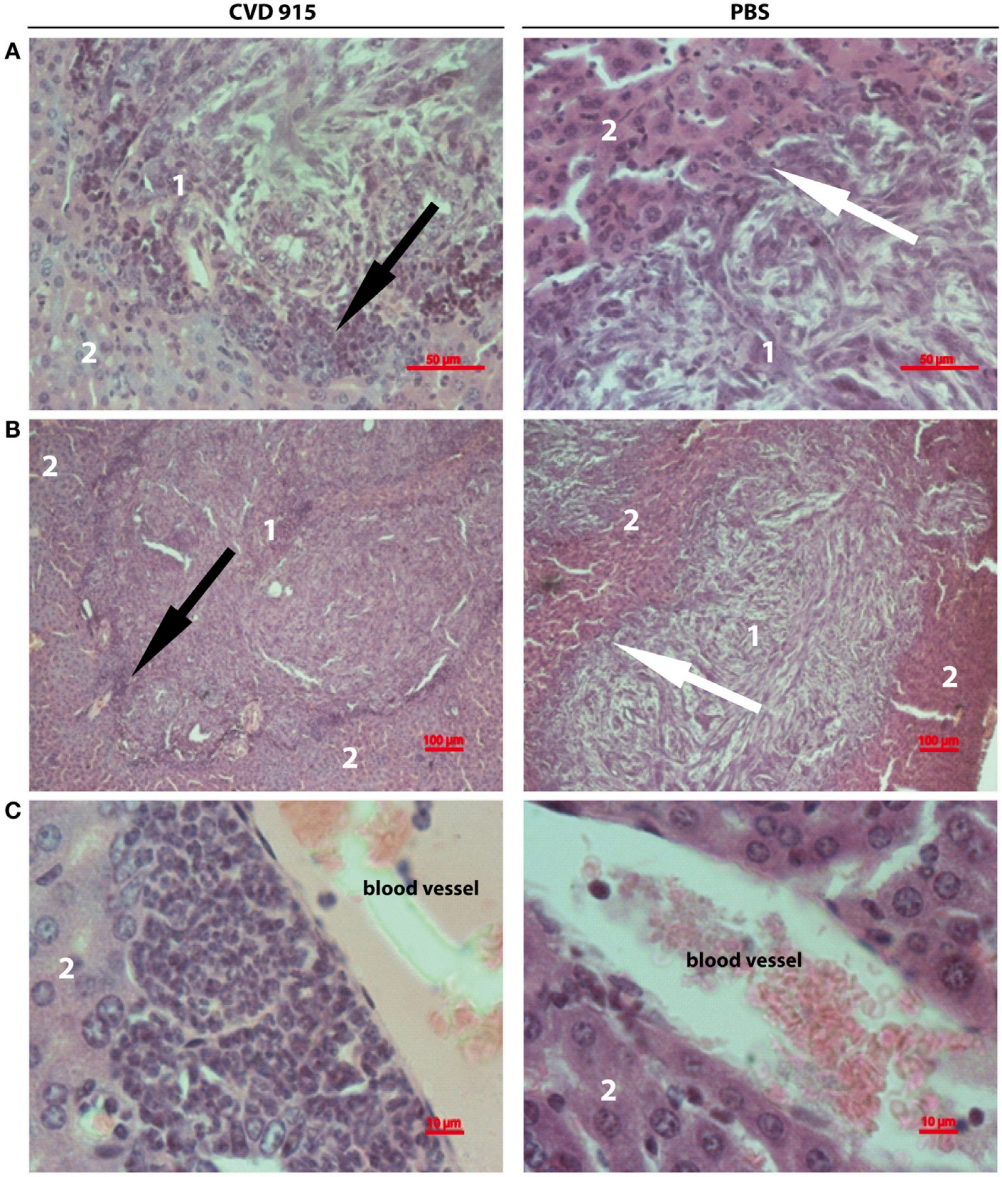
**Liver-infiltrating inflammatory cells after oral *Salmonella-*based treatment**. Twenty-one days after treatment, livers were removed from tumor-bearing mice. Images showing representative H&E stained liver sections from CVD 915- (left panels) or PBS-treated (right panels) animals. **(A,B)** Image of a metastatic focus in the liver. **(A)** Original magnification 400×. **(B)** Original magnification 100×. **(C)** Left: image of perivascular infiltrating leukocytes. Right: absence of perivascular infiltrate (original magnification 1000×). **(A–C)** Images shown are from one liver representative of five animals per group. (1) tumor nodule, (2) hepatic tissue, black arrows indicate leukocyte infiltration around a metastatic nodule, white arrows indicate the absence of leukocyte infiltration around a metastatic nodule.

A comprehensive histopathologic analysis of livers from tumor-bearing mice 21 days after treatment was performed. CVD 915-treated mice exhibited livers with large leukocyte infiltration, including lymphocytes, monocytes, and neutrophils surrounding tumor nodules. In contrast, a poor infiltrate was seen in only some untreated livers (Figures 6A,B). Livers from *Salmonella-*treated mice were highly infiltrated by leukocytes in the perivascular tissue (Figure 6C), whereas most control livers exhibited poorly infiltrated. These results complement flow cytometric immunophenotype data, providing additional information about the location of the leukocytic infiltrate induced by *Salmonella*.

## Discussion

One of the promising strategies in cancer immunotherapy is to use the power of the efficient immune response against microbial agents to fight malignancy. The liver is a particular immunological site in which tolerance mechanisms prevail over an immune-reactive state (9). Thus, breaking the hepatic tolerance and counteracting the immunosuppressive mechanisms induced by cancer cells within the liver are required to induce an effective immune response against liver cancer. In this study, we demonstrate the ability of the CVD 915 *Salmonella*-based vaccine to reduce the development of liver metastases from breast cancer cells by promoting a substantial reduction of the overall hepatic tumor burden in *Salmonella*-treated mice. This result is in agreement with preliminary studies from our group showing that this treatment can elicit similar therapeutic response against hepatic metastases in a mouse model of liver metastases from T-cell lymphoma (data not shown). The liver is a common target of cancer metastases. The fact that *Salmonella*-based vaccines prove to be useful against breast and lymphoma metastases implies that they might be suitable to treat metastases from other types of cancer. Our findings correlate with other studies in which orally attenuated *Salmonella* Typhimurium was tested in a mouse model of liver metastases from colon adenocarcinoma (22, 23). Based on our results, orally attenuated *Salmonella*-based vaccination could be used in patients as a neoadjuvant therapy before the resection of the primary tumor, especially to prevent the development of liver metastases. It could be also speculated that the use of human-restricted, attenuated strains of *S*. Typhi should be more appropriate to achieve a wider dissemination throughout the body, thus favoring tumor colonization by bacteria in humans. Future clinical trials in cancer patients might prove this hypothesis.

Previous reports have proposed the resection of liver metastases from breast cancer in order to improve survival in patients with solitary liver metastases (24). In addition, it was noted that the numbers and sizes of liver tumor nodules are crucial factors for success in this therapy (25). The significant reduction in the number and volume of liver metastases achieved with *Salmonella*-based immunization, although not curative, might enhance patient survival and increase the chances of success after liver surgery.

Unlike humans, mice are resistant to oral infection with *S*. Typhi (26, 27). Because CVD 915 is an auxotrophic strain of *S*. Typhi, it should be less capable of *in vivo* multiplication in the mouse than a wild-type strain (28). This issue could explain the absence of liver colonization by the attenuated strain CVD 915. *Salmonella* antigens could spread to the liver via the portal vein like those from the intestinal bacterial flora (11). DCs in the gastrointestinal tissue have a unique morphology, which is characterized by extended transepithelial dendrites. These processes allow DC to sample antigens directly from the lumen (29) and drive them to the liver. Liver DC circulate through hepatic sinusoids toward lymph draining vessels and undergo an increase in expression levels of MHC II molecules and IL-12 upon maturation (30). It could be also hypothesized that antigen-loaded DC and/or exosomes from bacterial-infected macrophages recruited into the liver after treatment, act as antigen presenting cells to prime specific T cells in the liver and/or LDLN (31, 32).

Several authors have already studied the immunogenicity of live *S*. Typhi vaccines administered by mucosal routes in mouse models (33–35), especially humoral or cellular immune responses in blood and spleen. However, the immune response to these vaccine strains in the liver and its main draining lymph nodes (celiac and portal nodes) has not been documented so far. In this study, we demonstrate that a strain of *S*. Typhi provokes a strong innate immune response in the liver, which is characterized by a huge increase in the IFN-γ production by IHIC and augmented DC and CD4^+^ T cell populations in celiac and portal LDLN. Other authors have demonstrated that mice infected with *Salmonella* Typhimurium develop a Th1 response characterized by the production of large amount of IFN-γ, mainly by the IHIC (36). Further, Zhang et al. identified hepatic NK cells as the principal IFN-γ-expressing lymphocyte upon acute *E. coli* challenge (10), although NKT and conventional T cells were also involved in IFN-γ production. Thus, NK is probably the most important source of IFN-γ in the liver of *Salmonella*-treated mice. Further studies should be performed in order to elucidate the role of these cell populations as early effectors of innate immunity activated by *Salmonella* in our liver metastases model. Collectively, our findings provide first evidence for an involvement of the liver-draining celiac and portal lymph nodes in the hepatic immune response against oral *Salmonella* spp.

Although humoral immunity is desirable for acute infections, antitumor Th1-type cellular immunity is responsible for eradicating established cancers (37). We detected an increased frequency in the CD4^+^ cell population and a decrease in the B220^+^ cell population (mostly B cells) both in LDLN and liver from treated mice. These results are consistent with a predominant T-cell-mediated immune response located at the tumor site and LDLN. Besides, it is broadly known that IFN-γ is a critical component of the immune system in antitumor immune responses (38). Here, we demonstrated a huge increase in production of Th1-type cytokines (including IFN-γ, TNF, and IL-2) by IHIC and LDLN cells in *Salmonella*-treated animals. Moreover, IFN-γ and TNF levels produced by LDLN increased in the presence of LM3 cells, thus indicating a tumor-specific cellular immune response. Furthermore, a tumor-specific TNF production by IHIC was found in immunized mice. By contrast, IL-2 levels decreased upon *in vitro* tumor-stimulation of lymphoid cells from *Salmonella*-treated mice. The decline of IL-2 levels could be due, at least in part, to its consumption by antigen-induced IL-2α receptor^+^ (CD25^+^) cells, such as activated tumor-specific CD4- and CD8-T lymphocytes (39). Since IFN-γ and TNF exert synergic cytotoxic effects on endothelial and tumor cells (40), the presence of these Th1-type cytokines in the liver microenvironment, the tumor site, could significantly contribute to the antitumor effects promoted by oral *Salmonella*-based immunotherapy. However, we cannot rule out the involvement of Th2- and/or Th17-type cytokines in the antitumoral immunity induced by oral *Salmonella*-based vaccination.

The IFN-γ expression by CD8^+^ cells is a reliable functional surrogate for the identification of cytotoxic T lymphocytes (41). Herein, we demonstrate an increased percentage of tumor-specific CD8^+^IFN-γ^+^ T cells in the LDLN from *Salmonella*-treated mice. These data show that oral *Salmonella*-based vaccination breaks liver immunosuppression, thus favoring the development of a tumor-specific immune response in LDLN and, probably, the migration of effector CD8^+^IFN-γ^+^ T cells toward the liver. It can be also noted that a higher leukocytic infiltrate surrounding liver tumor nodules was found in *Salmonella*-treated mice, which suggests a positive response to therapy (42). Evidence from clinical studies indicates that Tregs have well-defined roles in suppressing antitumor immunity [i.e., through a negative feedback on effector T cells (43)]. Also, Tregs are important in maintaining liver immunological self tolerance (44). Based on these data and our previous findings demonstrating that CVD 915-based immunotherapy reduce Tregs in tumor-draining lymph nodes in mice (6, 7), it can be speculated that this oral *Salmonella*-based vaccination will also be able to promote a decrease in the Treg cell population in the liver and/or LDLN, thus supporting the generation of an efficient tumor-specific cellular immunity.

Lymph nodes have a key role in host defense against pathogens and also, tumors. This study provides new insight into the critical role of main liver-draining lymph nodes (celiac and portal nodes) in the cellular immune response against hepatic tumors, which resulted in the elimination of liver metastases in LM3 tumor-bearing mice after oral *Salmonella*-based vaccination. In contrast, MLN nodes appeared to play a secondary role. These results agree with previous data from Barbier et al. (12). These authors demonstrated that celiac and portal nodes are the preferential site of DC migration and T cell activation in mice. However, they rarely detected the activation of T cells in the first mesenteric node. Interestingly, our findings will further provide valuable insights for understanding the immune response to hepatic tumors and for performing appropriate preclinical validation of new anti-liver cancer therapies, including immunotherapies.

In conclusion, this study demonstrates that an oral live attenuated *S*. Typhi vaccine strain can elicit a successful immune response, which is capable of preventing the development of liver metastases. The early type-1 innate immunity activated by a single oral dose of a *S*. Typhi is sufficient to break not only liver-inherent immune tolerance but also tumor-induced immunosuppression. The proinflammatory microenvironment elicited in the liver by oral *Salmonella* triggers a tumor-specific Th1-type immune response, which efficiently reaches the liver and its recently identified draining lymph nodes rendering a drastic reduction of hepatic metastases. The proven antitumor efficacy of oral *S*. Typhi vaccination encourages future clinical evaluation of this simple and inexpensive immunotherapy as a neoadjuvant and/or adjuvant treatment for liver metastatic cancer.

## Author Contributions

Conception and design: AV and CW. Execution of experiments: AV and AT. Acquisition of data: AV, MG, AT, and AC. Analysis and interpretation of data: AV, CM, MG, JG, and CW. Drafting of the manuscript: AV and CW. Critical revision of the manuscript: MG, AC, JG, and CM. Obtained funding: AV, CM, and CW. Study supervision: CW. Final approval of the version to be published: AV, CM, MG, AT, AC, JG, and CW. Agreement to be accountable for all aspects of the work: AV, CM, MG, AT, AC, JG, and CW.

## Conflict of Interest Statement

The authors declare that the research was conducted in the absence of any commercial or financial relationships that could be construed as a potential conflict of interest.

## Abbreviations

DBW, differential body weight; IHIC, intrahepatic immune cells; LDLN, liver-draining lymph nodes; *S*. Typhi, *Salmonella enterica* serovar Typhi.
