# Supplementary figures and images for: An Oral Salmonella-Based Vaccine Inhibits Liver Metastases by Promoting Tumor-Specific T-Cell-Mediated Immunity in Celiac and Portal Lymph Nodes: A Preclinical Study
Source: Front Immunol. 2016 Mar 1;7:72. doi: 10.3389/fimmu.2016.00072 (PMC4771756; doi:10.3389/fimmu.2016.00072)

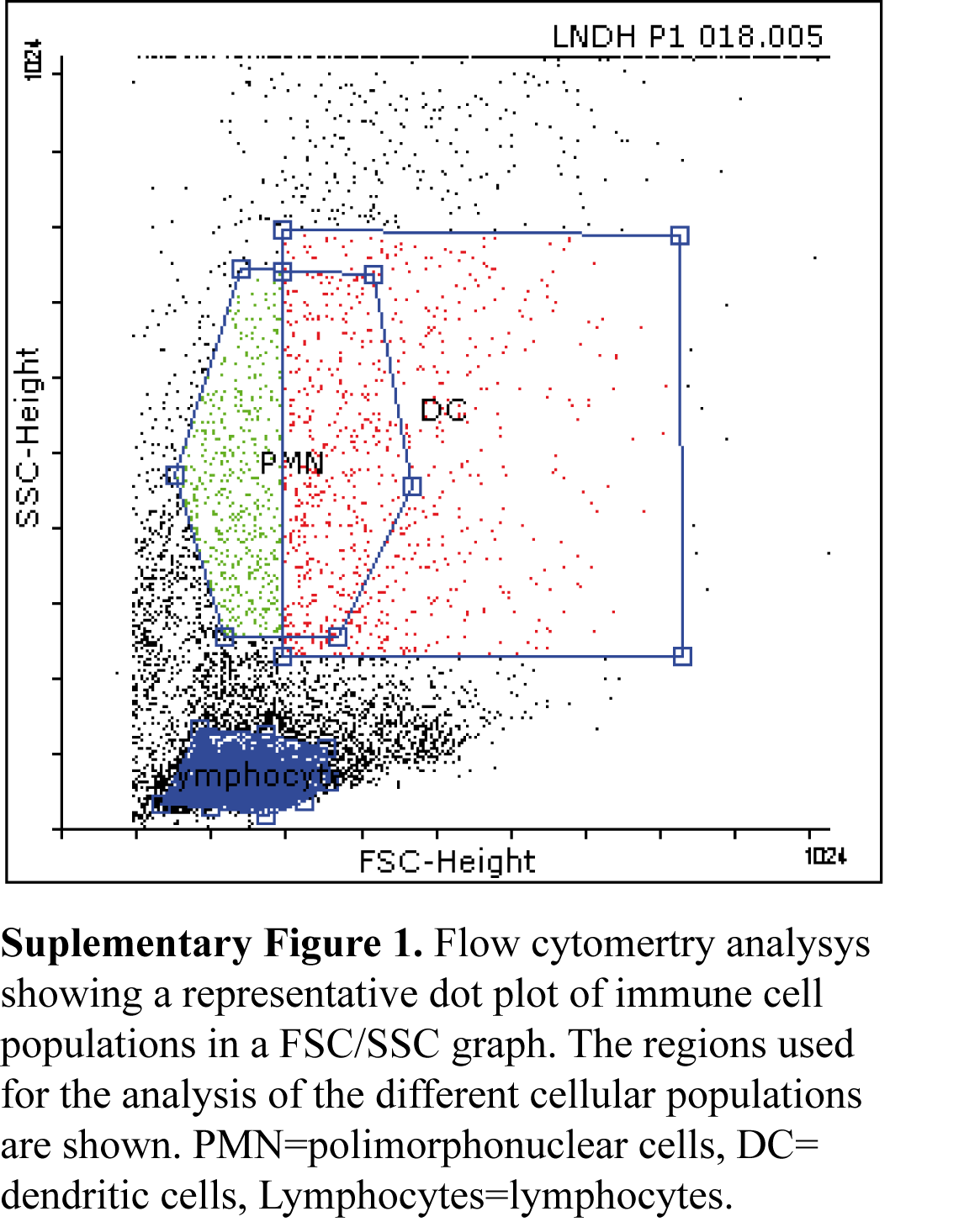

Supplement: Supplementary file 1 [file Image_1.TIF]

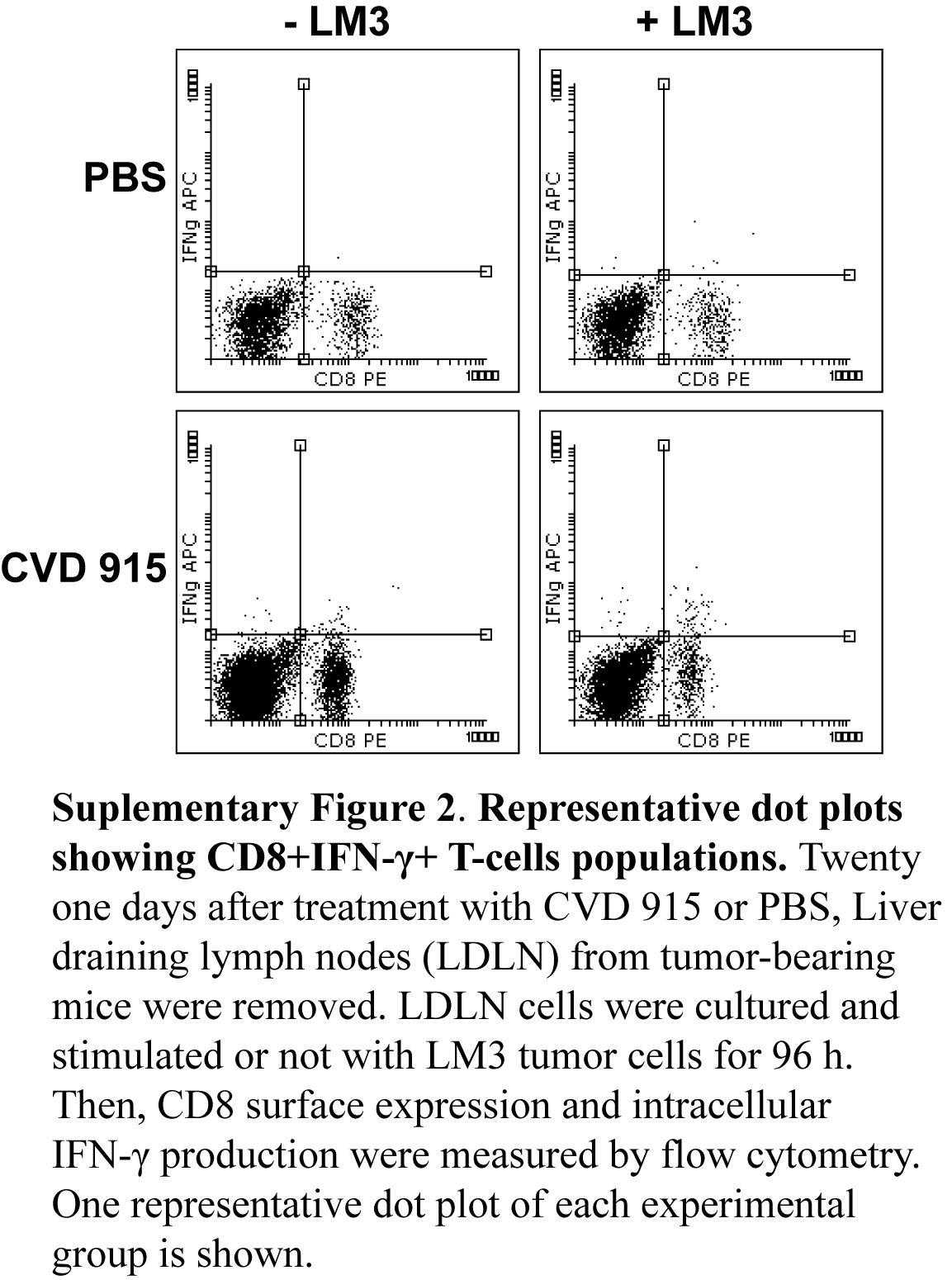

Supplement: Supplementary file 2 [file Image_2.TIF]
